# Status Quo and Trends of Intra-Arterial Therapy for Brain Tumors: A Bibliometric and Clinical Trials Analysis

**DOI:** 10.3390/pharmaceutics13111885

**Published:** 2021-11-06

**Authors:** Julian S. Rechberger, Frederic Thiele, David J. Daniels

**Affiliations:** 1 Department of Neurologic Surgery, Mayo Clinic, Rochester, MN 55905, USA; Daniels.David@mayo.edu; 2 Mayo Clinic Graduate School of Biomedical Sciences, Mayo Clinic, Rochester, MN 55905, USA; 3 Department of Neurology, Mayo Clinic, Rochester, MN 55905, USA; Thiele.Frederic@mayo.edu; 4 Department of Molecular Pharmacology and Experimental Therapeutics, Mayo Clinic, Rochester, MN 55905, USA

**Keywords:** brain tumor, glioma, drug delivery, injection, intra-arterial, chemotherapy, targeted therapy, immunotherapy, nanoparticles, treatment

## Abstract

Intra-arterial drug delivery circumvents the first-pass effect and is believed to increase both efficacy and tolerability of primary and metastatic brain tumor therapy. The aim of this update is to report on pertinent articles and clinical trials to better understand the research landscape to date and future directions. Elsevier’s Scopus and ClinicalTrials.gov databases were reviewed in August 2021 for all possible articles and clinical trials of intra-arterial drug injection as a treatment strategy for brain tumors. Entries were screened against predefined selection criteria and various parameters were summarized. Twenty clinical trials and 271 articles satisfied all inclusion criteria. In terms of articles, 201 (74%) were primarily clinical and 70 (26%) were basic science, published in a total of 120 different journals. Median values were: publication year, 1986 (range, 1962–2021); citation count, 15 (range, 0–607); number of authors, 5 (range, 1–18). Pertaining to clinical trials, 9 (45%) were phase 1 trials, with median expected start and completion years in 2011 (range, 1998–2019) and 2022 (range, 2008–2025), respectively. Only one (5%) trial has reported results to date. Glioma was the most common tumor indication reported in both articles (68%) and trials (75%). There were 215 (79%) articles investigating chemotherapy, while 13 (65%) trials evaluated targeted therapy. Transient blood–brain barrier disruption was the commonest strategy for articles (27%) and trials (60%) to optimize intra-arterial therapy. Articles and trials predominately originated in the United States (50% and 90%, respectively). In this bibliometric and clinical trials analysis, we discuss the current state and trends of intra-arterial therapy for brain tumors. Most articles were clinical, and traditional anti-cancer agents and drug delivery strategies were commonly studied. This was reflected in clinical trials, of which only a single study had reported outcomes. We anticipate future efforts to involve novel therapeutic and procedural strategies based on recent advances in the field.

## 1. Introduction

Conventional treatment options for brain tumors rely on surgery, radiotherapy, and systemic pharmacotherapy. Oral and intravenous drug administration is often associated with poor brain distribution and bioavailability, limiting therapeutic effect, and contributing to unsatisfactory clinical outcomes [1,2,3,4,5,6]. High-grade gliomas, including glioblastoma and H3K27-altered diffuse midline glioma, with a median survival of approximately 12–15 months after diagnosis, stand a grim example of this failure to develop effective treatments [7,8,9,10,11]. In this multiomics era of biomedical research, insights into biological aspects of cancer have allowed us to identify potential targets that could improve the clinical course of these devastating diseases [12,13,14,15]. The first-pass effect and the blood–brain barrier (BBB), however, remain significant obstacles for therapeutic access to the brain and hinder novel therapies from unfolding pharmacologic potential [16,17,18,19,20].

One proposed solution to overcome these hurdles comprises strategies to minimize systemic drug exposure and modulate the BBB, which could expand the spectrum of usable drugs and potentially improve therapeutic efficacy and tolerability. Intra-arterial injection into intracranial vessels is one such strategy, with the potential to increase drug responses to primary and metastatic brain tumors [21,22,23,24,25,26,27,28,29,30]. Intra-arterial infusion of anti-cancer therapies can be combined with concurrent administration of a variety of agents, including chemical reagents, penetration drug carriers, or microbubbles for focused ultrasound, to selectively open the BBB in areas of interest [17,25,31,32]. By accessing intracranial vessels through peripheral arteries and directly administering BBB-disrupting and therapeutic agents into the arterial supply to the brain, intra-arterial injection facilitates greatly improved local drug delivery, increased intra-tumoral concentration, and lowered systemic exposure [33,34,35,36,37].

Since it was first described more than half a century ago, there have been considerable efforts not only to explore the biological mechanisms behind intra-arterial therapy but also to evaluate its applicability to a wide range of diseases. To date, multiple research studies are quoted to have investigated intra-arterial drug administration, yet there has been little, if any, translational impact observed for brain tumors [31,34,38,39,40,41,42,43]. Therefore, it is important to characterize how impactful the literature and previous clinical trials have been to predict where this drug delivery approach is heading. The aim of this study was to analyze the bibliometric parameters of available articles and evaluate registered clinical trials that have incorporated intra-arterial drug injection as a treatment strategy for brain tumors. This will provide a profile of the most impactful articles and trials to better inform clinicians of the current research landscape of intra-arterial drug delivery. Furthermore, this will enable future clinical trials to optimize and justify their design based on previous experiences to maximize trial discoveries and outcomes.

## 2. Methodology

The search strategy was designed to capture all possible Scopus-indexed articles and ClinicalTrials.gov-registered clinical trials referring to intra-arterial therapies for the treatment of brain tumors. Elsevier’s Scopus facilitates access to peer-reviewed articles from approximately 22,000 journals. It offers one of the largest scientific literature capture reaches of biomedical electronic research databases [44]. ClinicalTrials.gov is a database provided by the US National Library of Medicine that contains referenced clinical trials on a wide range of conditions and diseases conducted around the world. It has been shown to have entries on 388,133 research studies from all 50 states of the USA and 219 countries worldwide [45,46]. Both databases were searched and screened independently by two investigators (J.S.R. and F.T.). We searched Scopus for referenced articles from its date of inception to August 2021 using the following string of search terms: (intra-arterial) AND (therapy OR treatment) AND (brain tumor OR glioma). The ClinicalTrials.gov portal was searched in August 2021 using “brain tumor”, “glioma”, and “intra-arterial injection” search terms for Condition or disease and Intervention/treatment, respectively. Any discrepancies were resolved by discussion until consensus was reached. Publications were limited to the English language.

To be included in our subsequent analyses, articles and clinical trials were required to investigate (1) intra-arterial administration of (2) therapeutics as (3) a treatment strategy for (4) tumors related to (5) the brain. In the case of articles and research studies that explored intra-arterial injection as a purely diagnostic tool, focused on diseases other than primary or secondary brain tumors, or investigated tumors of other organ systems, these were not included due to lack of specificity. Assessment of articles and trials to satisfy these criteria was performed independently by two investigators (J.S.R and F.T.), with any discrepancies resolved by discussion. There was no location restriction for eligible database entries.

The following validated article variables were then extracted from the Scopus database: article title, year, authors, number of authors, country of correspondence of the senior author, journal, Scopus citations, document type, study type, tumor type, therapy type, and type of treatment strategy for optimizing intra-arterial administration. Regarding the latter variable, 5 categories were defined: (1) nanoparticles, (2) transient BBB disruption, (3) transient cerebral hypoperfusion or flow arrest, (4) superselective intra-arterial cerebral infusion, and (5) the combination of imaging techniques with intra-arterial infusion of contrast agents or labeled therapeutic agents. With respect to study type, articles were dichotomized to be either basic science (BSc) or clinical (CL). BSc articles were ones primarily describing nonpatient investigations, such as in vitro and in vivo models, whereas CL articles were ones focusing on patient outcomes, including feasibility, safety, and survival. Clinical trial outcomes extracted from ClinicalTrials.gov included National Clinical Trial (NCT) number, title, sponsor, institution of correspondence, country of origin of the corresponding institution, number of institutions involved, involvement of outside countries, status, availability of results, type of condition, type of primary intervention, primary and secondary outcome measurements, gender enrollment, age of enrollment, number of patients enrolled, study phases, study type, start year, completion year, year of the first release of results, and last updated year [47]. Missing data were denoted as “not reported”. All data analyses, including the generation of figures and tables, were performed using Pandas 1.3.2 (i.e., Python Data Analysis Library), an open-source data analysis and manipulation tool that is built on top of the Python programming language [48]. No statistical comparisons were conducted.

## 3. Results

### 3.1. Article Characteristics

**Figure 1:**
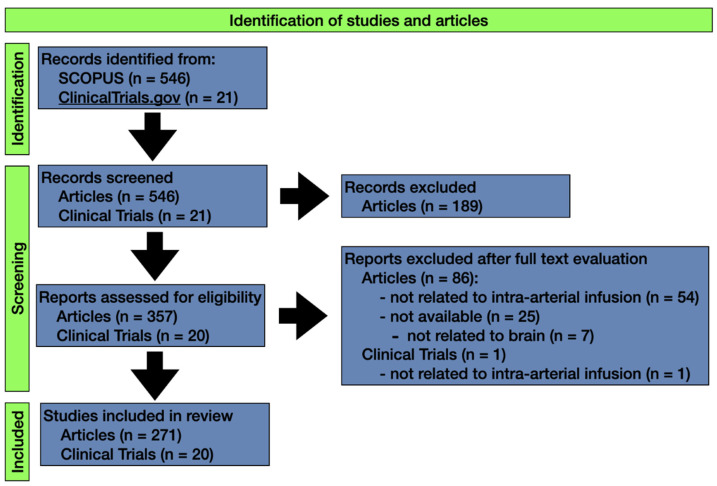
Methodological approach to identify articles and clinical trials on intra-arterial brain tumor therapy via databases and registers.

A total of 546 articles were retrieved from Scopus after the initial database search. We screened titles and abstracts to obtain 357 articles not meeting any exclusion criteria. Full-text evaluation yielded 271 articles that were finally included in our study (Figure 1). A summary of the whole article cohort is provided in Table 1, and detailed results can be found in Tables S1–S13, Supplementary Materials. We identified 227 (84%) as original articles and 44 (16%) as review articles. There were 70 BSc articles (26%) and 201 CL articles (74%). Fifty-four (20%) were published open access, and therefore freely accessible online (Table S1). The most common articles for intra-arterial drug delivery in brain tumors were for gliomas (*n*= 184, 68%), including glioblastoma, gliosarcoma, diffuse intrinsic pontine glioma and glioma without further specification, brain metastasis (*n* = 12, 4%), and lymphoma (*n* = 5, 2%). Sixty-six articles reported inclusion of multiple tumor types (24%) (Table S2).

The median citation count was 15 (range, 0–607), with the most-cited article to date a review by Hochberg et al. [49], published in 1988 with 607 citations (“Primary central nervous system lymphoma” in the *Journal of Neurosurgery*). The most cited original article was the CL study by Doolittle et al. [34], published in 2000 with 300 citations (“Safety and efficacy of a multicenter study using intraarterial chemotherapy in conjunction with osmotic opening of the blood–brain barrier for the treatment of patients with malignant brain tumors” in Cancer). Matsukado et al. [50] published in 1996 the most-cited BSc article with 151 citations (“Enhanced tumor uptake of carboplatin and survival in glioma-bearing rats by intracarotid infusion of bradykinin analog, RMP-7” in Neurosurgery) (Table S3).

With regard to contributing authors, the median number of authors for original and review articles was five (range, 1–18). The most authored article was the original, CL study by Angelov et al. [51], published in 2009 with 18 authors (“Blood–brain barrier disruption and intra-arterial methotrexate-based therapy for newly diagnosed primary CNS lymphoma: A multi-institutional experience” in the *Journal of Clinical Oncology*). The highest number of authors for BSc articles was 12: Liu et al. [52] published their manuscript in 1991 (“Effects of intracarotid and intravenous infusion of human TNF and LT on established intracerebral rat gliomas” in Lymphokine and Cytokine Research) whereas the article by Mao et al. [53] was published in 2020 (“Peritumoral administration of IFNβ upregulated mesenchymal stem cells inhibits tumor growth in an orthotopic, immunocompetent rat glioma model” in Journal for ImmunoTherapy of Cancer). The most authored review article was by Aoki et al. [54], published in 1993 with 13 authors (“Supraophthalmic chemotherapy with long tapered catheter: Distribution evaluated with intraarterial and intravenous Tc-99m HMPAO” in Radiology). The authors with the most senior-authored articles overall were E.A. Neuwelt and J.A. Boockvar, who both contributed eight articles [34,36,37,51,55,56,57,58,59,60,61,62,63,64,65,66] (Table S4).

**Figure 2:**
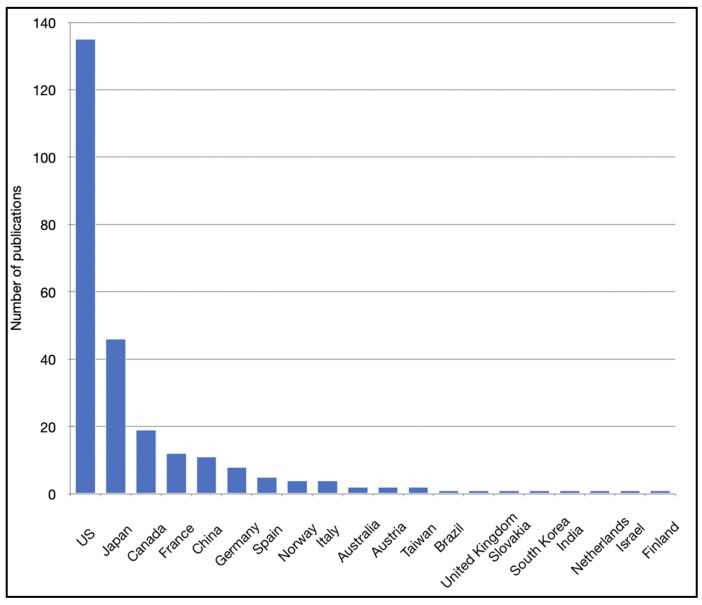
Distribution of articles about intra-arterial drug delivery for the treatment of brain tumors based on the country of correspondence.

All articles were published between 1962 and 2021 (Figure 2), with a median of 5 publications per year. The peak year (median) with most-published articles was 17 (6%) articles published in 1986. Original articles and reviews peaked with respect to their annual publication number in 1986 and 2020, respectively. Most BSc articles were published in 1999, while CL articles had their peak year in 1986 (Table S5).

**Figure 3:**
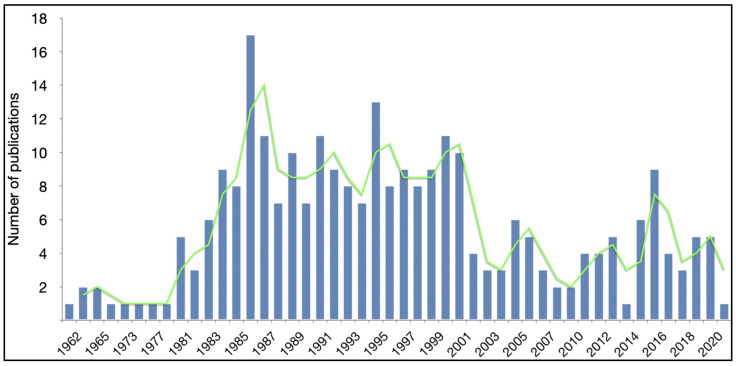
Distribution of articles about intra-arterial drug delivery for the treatment of brain tumors based on the year of publication.

A total of 20 countries were denoted as the location for correspondence of all articles (Figure 3). The USA was the country with the highest contribution, with 135 articles (50%), followed by Japan and Canada, with 46 (17%) and 19 (7%), respectively. The USA was the most common country of correspondence for all document and study types (Table S6).

One hundred and twenty journals contributed to articles of intra-arterial therapy for the treatment of brain tumors. The most common ones were the *Journal of Neuro-Oncology*, with 48 (18%) articles, the *Japanese Journal of Cancer and Chemotherapy* (*n* = 14, 5%), and *Neurosurgery* (*n* = 13, 5%). The journal publishing most original studies, review articles, BSc articles, and CL articles was the *Journal of Neuro-Oncology* (Table S7).

**Figure 4:**
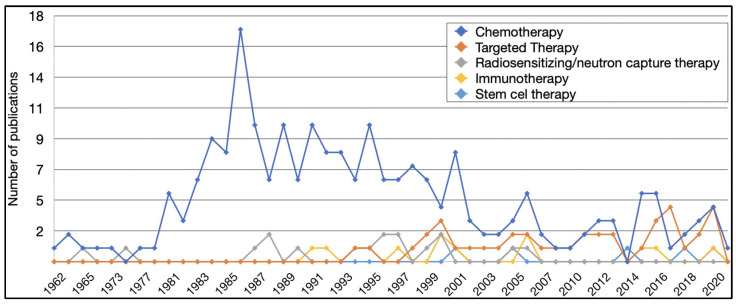
Different therapy types investigated in articles of intra-arterial drug delivery for the treatment of brain tumors based on the year of publication.

In terms of therapy types used with intra-arterial delivery, general chemotherapy was the most common, with 215 (79%) articles, followed by targeted therapy (*n* = 40, 15%), and radiosensitizing or neutron capture therapy (*n* = 17, 6%). The number of articles per therapy type per year of publication is illustrated in Figure 4. Chemotherapy was the top therapeutic strategy in all but three years (1973, 2014, and 2017). The most commonly studied chemotherapeutic agents included i.a. carmustine (*n* = 29, 13%), i.a. nimustine (*n* = 20, 9%), and i.a. cisplatin (*n* = 23, 11%). Twenty-three articles (11%) mentioned the general concept of intra-arterial chemotherapy without further specification (Tables S8–S12).

**Figure 5:**
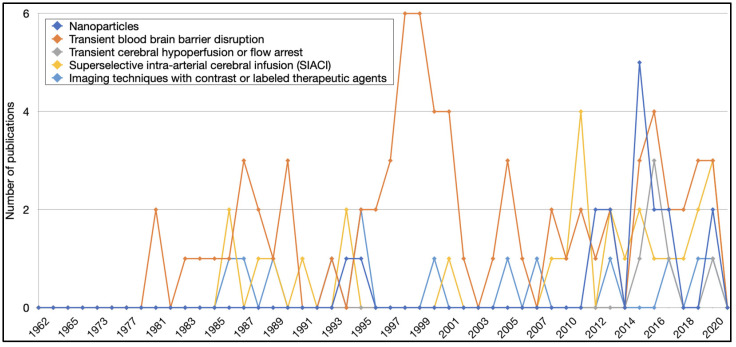
Treatment strategies to optimize intra-arterial drug delivery for the treatment of brain tumors in pertinent articles based on the year of publication.

At least 1 additional treatment strategy for optimizing intra-arterial drug delivery was evaluated in 104 articles (Figure 5). The most common strategy was transient BBB disruption, mentioned in 74 articles (27%). Transient BBB disruption was followed by superselective intra-arterial cerebral infusion (*n* = 27, 10%), and nanoparticles (*n* = 17, 6%). Among BBB-opening modalities, mannitol was the most common one, referenced in 48 articles (65%), followed by bradykinin/RMP-7 (*n* = 16, 22%) (Table S13).

### 3.2. Clinical Trial Characteristics

The initial search of the ClinicalTrials.gov portal yielded 21 clinical trials for screening. One trial was excluded because it did not investigate intra-arterial drug injection, but rather looked at cerebral blood perfusion changes during emergence from general anesthesia for craniotomy using an intra-arterial pressure line [67]. Consequently, 20 trials were included in our study, all of which were interventional in nature. Included trials have been summarized in Table 2, with individual details listed in Tables S14–S24.

Glioblastoma was the most common brain tumor indications for trials involving intra-arterial drug delivery (*n*= 13, 65%), followed by anaplastic astrocytoma (*n* = 8, 40%) (Table S14). The median commencement year was 2011, with trials reporting start dates between 1998 and 2019. As for expected completion year, the median was 2022 (range, 2008–2025) (Table S15). As of August 2021, 6 (30%) trials are reported to have completed recruiting patients, 8 (40%) are still recruiting, and 2 (10%) are active but not recruiting. Two (10%) trials are declared as suspended (Table S16).

All trials reported target enrollment sizes between 3 and 60 patients, with the Portland-based trial “NCT00075387: Combination Chemotherapy With or Without Sodium Thiosulfate in Preventing Low Platelet Count While Treating Patients With Malignant Brain Tumors” [68] targeting the most (*n* = 60). This trial has an estimated study completion date in spring 2023 (Table S17). With regard to age of enrollment, the median minimum patient age was 18 years (range, 1 month–18 years), with 18 (90%) of trials using this threshold. The median maximum patient age was 99 years (range 17–120 years), with 2 (10%) trials focusing solely on the pediatric demographic while 18 (90%) also included adult patients (Table S18).

Phase category was reported for all clinical trials included in this study. With 9 (45%) trials, phase 1 was the most common phase design. Eight (40%) trials were registered as both phase 1 and 2. A total of 3 (15%) trials were exclusively phase 2. Two phase 2 studies were randomized. Allocation was non-randomized in 4 phase 1 trials, while allocation type was not available for all other trials (Table S19).

In terms of the different types of primary intervention, therapeutic drug alone was the most common, with 14 (70%) trials. Combinations of radiation and drug therapy as well as therapeutic drug and psychological assessments were applied in 2 (10%) trials each. Investigations of biological agents alone (*n* = 1, 5%) and in combination with drug therapy (*n* = 1, 5%) were also reported (Table S20). Overall, there were 20 different types of primary intervention combinations evaluated in clinical trials of intra-arterial therapy for brain tumors (Table S21). Based on our original classification, targeted therapy was the therapeutic strategy most commonly investigated (*n* = 13, 65%), followed by chemotherapy (*n* = 8, 40%). Of these, i.a. bevacizumab (*n* = 5, 25%), i.a. cetuximab (*n* = 3, 15%), and i.a. melphalan (*n* = 2, 10%) were most common (Table S22). Transient BBB disruption was used in 12 (60%) trials. Thirteen (65%) trials explored superselective intra-arterial cerebral infusion as a strategy to optimize intra-arterial administration (Table S23).

Feasibility, safety, and toxicity of a treatment or intervention was the most common primary outcome reported (*n* = 11, 55%). With respect to other primary outcomes, 6 (35%) trials reported progression-free survival and 5 (25%) reported overall survival. One study (5%) reported tumor response and intracellular carboplatin accumulation as primary outcome measurement. The most common secondary outcome reported was progression-free survival 11 (55%), followed by feasibility, safety, and toxicity (*n* = 9, 45%) and overall survival (*n* = 8, 40%) (Table S24).

As of August 2021, only 1 (5%) trial has posted results (NCT00362817: Carboplatin and Temozolomide (Temodar) for Recurrent and Symptomatic Residual Brain Metastases) [69]. This study started recruiting patients in 2004 and reported results in 2015. Seventeen patients older than 18 years, who had all received prior systemic chemotherapy for primary cancers in parts of the body other than the brain, were enrolled to investigate the use of intra-arterial carboplatin and oral temozolomide for the treatment of recurrent and symptomatic residual brain metastases. In terms of primary outcome, the reported response rate, evaluated by MRI criteria (MacDonald criteria), was approximately 43%. Secondary outcome measures included 25 weeks overall survival, 23 weeks progression-free survival, and no incidence of CNS toxicities or CNS tumor-related deaths. In 7 (41%) cases, systemic disease progression was determined as cause of death (Table 3).

A total of 10 different institutions coordinated all 20 clinical trials on intra-arterial therapeutic delivery to brain tumors. Three different countries were listed as the location for correspondence of all trials, with the USA contributing 18 (90%) trials. The Lenox Hill Brain Tumor Center, located in New York City, coordinated the most trials (*n* = 10, 50%) [70,71,72,73,74,75,76,77,78,79]. The only other institution coordinating more than one trial was the OHSU Knight Cancer Institute in Portland, OR (*n* = 3, 15%) [68,80,81]. Only two trials had corresponding institutions outside the USA, with the Beijing YouAn Hospital (China) and the Centre hospitalier universitaire de Sherbrooke (Canada) both coordinating one (5%) trial [82,83] (Table S25). The median number of institutions involved was one (range, 1–3), with 18 (95%) studies involving a single institution. All trials were conducted in a single country.

## 4. Discussion

The intention of this study was to identify and characterize the published literature and registered clinical trials on intra-arterial drug administration for brain tumor treatment. We identified 271 articles and 20 trials to meet our inclusion criteria. These numbers are reflected in the quoted numbers reported by recently published reviews [31,84], even though this is the first study to offer a precise number of clinical trials involving intra-arterial brain tumor therapy, highlighting a previously unreported area in the field as to how many distinct trials have officially been registered and conducted since the technique was first described in 1950 [85]. The complexity of successfully translating intra-arterial drug delivery into the clinic is demonstrated by the fact that in the last 20 years, only 6 trials eligible for this review have completed recruitment [69,74,76,77,82,86], and results of just a single study are publicly available at the ClinicalTrials.gov portal as of August 2021 [69]. Despite these findings, given the discovery of novel biological and molecular features of brain tumors potentially amenable to therapy [12,13,87,88], we posit that more tumor-specific intra-arterial interventions will emerge in future trials to add to the current body of research studies.

The development of intra-arterial technologies was historically driven by the need to minimize the systemic toxicity of traditional anti-cancer agents and propelled by advances in endovascular techniques; however, very few studies took into consideration the pharmacokinetic characteristics underlying intra-arterial drug delivery [89]. Although the neuro-oncological application of intra-arterial technology has been established by impactful CL articles [34,38,85], accurate and reliable pharmacological models to optimize the method, rate, and duration of drug injection for high local extraction and systemic clearance may be lacking to date [90]. Furthermore, biological hurdles to intra-arterial therapy of brain tumors, including the vascular heterogeneity within the tumor microenvironment, have to be considered in ongoing research efforts and future refinements [91]. The lack of effective therapies to be delivered by the intra-arterial route and reported in BSc articles could in part explain why CL articles and clinical trials remain without definitive success. Consequently, we expect to observe an increase in BSc articles in the future as our understanding of this technology and our ability to modify relevant drug properties continue to grow.

For those within the field of intra-arterial therapy for brain tumor treatment, it is not surprising that E.A. Neuwelt and J.A. Boockvar were identified as particularly impactful authors who pioneered the field and spearheaded recent advances of this drug delivery technique in terms of preclinical and clinical research. The portfolio of both these authors was predominantly focused on CL articles and clinical trials based in the USA. When considering all articles included in this study, E.A. Neuwelt was the author of most with 14 articles overall, of which he senior-authored 8 CL articles [34,51,55,56,57,58,59,60,92,93,94]. Through the Neuro-Oncology Blood-Brain Barrier Program at OHSU, he was also involved in initiating 3 clinical trials that are currently being conducted at the OHSU Knight Cancer Institute [68,80,81]. J.A. Boockvar authored 10 articles overall, serving as the corresponding author of 5 original and 3 review CL articles [35,36,37,61,62,63,64,65,66,95]. In his role as vice chair of neurosurgery at Lenox Hill Hospital, he has also been in charge of 10 clinical trials that were registered to evaluate intra-arterial brain tumor therapies, including bevacizumab, cetuximab, trastuzumab, and temozolomide alone or in combination with carboplatin, radiotherapy, and/or mannitol, for conditions such as glioblastoma, anaplastic astrocytoma, vestibular schwannoma, and brain metastasis [70,71,72,73,74,75,76,77,78,79]. Collectively, the clinical trials overseen by J.A. Boockvar and E.A. Neuwelt account for more than half of all trials included in this study. The general focus of both authors on translational research is largely reflected in the current research landscape of intra-arterial drug delivery for brain tumors, highlighting their significant impact on the field.

When Perese et al. [96] first proposed the intra-arterial route as a drug delivery strategy for treating patients with malignant brain tumors, they posited that this technology could be used to deliver large concentrations of a variety of anti-cancer agents to the brain without causing much systemic reaction. More than a half-century later, based on this study of pertinent articles and clinical trials, it appears their vision has, in part, been realized, but new hurdles have emerged. The largest indication for intra-arterial administration was chemotherapy, with 215 of 271 articles describing its BSc or CL use. Indeed, intra-arterial chemotherapy allowed relatively high dosing while minimizing systemic toxicity [41,59,63,64]. However, articles on chemotherapeutic drugs peaked over three decades ago, possibly indicating that these therapies were lacking efficacy with intra-arterial use. Furthermore, several articles alluded to chemotherapy-related safety concerns, some of which were unique to the intra-arterial delivery route [38,58,60,93]. This could explain why only a few traditional chemotherapeutics, all of which had previously demonstrated safety and efficacy in preclinical models and via other drug delivery strategies, have found their way to clinical trials. The fact that targeted therapy was the most commonly used therapy type in clinical trials could suggest a paradigm shift towards novel therapeutic strategies. Based on exciting developments in modern neuro-oncology, involving precision medicine [97], immunotherapy [98], and stem cell technology [99], we anticipate an increased number of articles and clinical trials investigating these therapies with intra-arterial injection in the future [100].

As effective treatment modalities for different types of brain tumors are desperately needed, it is not surprising that a number of technologies to improve the therapeutic effect of intra-arterial drug delivery have been proposed [24,30]. BBB penetrance, targeting, and accuracy of intra-arterial administration are of major interest, which is underscored by the high number of articles and clinical trials exploring strategies to account for these parameters. We found chemical reagents, and mannitol in particular, to be the oldest and most common approach to open the BBB. However, the capability of mannitol and newer agents, such as the bradykinin agonist RMP-7, to permeabilize the BBB is limited [50,63,101]. Focused ultrasound is a non-invasive strategy for disrupting BBB tight junctions in a reversible and controlled fashion and has the potential to be used with intra-arterial technologies [32,102,103]. The scientific complexity of this concept is underlined by the fact that of all 271 articles included in this study, only 2 review articles described focused ultrasound in combination with intra-arterial drug delivery [20,104]. Although mentioned by only a small proportion of articles, superselective intra-arterial cerebral infusion into the tumor-feeding arteries was reported in 13 of 20 clinical trials. This possibly indicates a trend towards using this advanced endovascular procedure to reduce neurotoxic side effects and ensure targeted intra-arterial therapy [35,36,37,61,63,64,65,95]. It is interesting to observe that several articles published in recent years investigated nanoparticles. The attraction of nanotechnology for intra-arterial drug delivery is multifold. Various authors noted these small particles could not only be loaded with different therapies, including small-molecule inhibitors, gene therapies or siRNAs, but could also be modified to cross the BBB through a variety of transport mechanisms and remain at the target site for longer periods of time to allow for a gradual release of loaded therapeutics [23,105,106,107,108,109,110,111,112,113].

We speculate that future refinements of intra-arterial brain tumor therapy will come from multiple angles. From a therapeutic perspective, studies to date are using mostly approved agents, as they are easier to translate; however, certain novel compounds may have superior pharmacokinetics and be ultimately more useful for intra-arterial drug delivery. Disease-specific drugs will enhance efficacy and minimize systemic and CNS-related side effects [87]. Drug modification or loading into nanoparticles can allow for improved BBB penetration, tumor targeting, and drug-tumor contact time of active compounds [114,115,116]. Labeled therapeutic agents as well as theragnostic nanoparticles have the potential to be used with advanced imaging techniques [50,106,109]. From a procedural point of view, optimizing currently available endovascular technologies and combining them with innovative strategies, such as superselective intra-arterial cerebral infusion or transient cerebral hypoperfusion/flow arrest, is crucial to ensure procedural safety and effect [31,61,89]. With regard to clinical trials, it will be important to move from phase 1 and 2 to phase 3 trials and to evaluate novel drugs and procedures in the context of randomized patient allocations. This will help to ensure the validity of conclusions not only from a feasibility and safety perspective but also in terms of efficacy. The inability of the vast majority of clinical trials to report results suggests that a collaborative approach may be necessary to improve the fate of future trials. Therefore, we stress the importance of intra- and inter-institutional collaboration in preclinical and clinical research for progress within the field of intra-arterial brain tumor therapy.

The intra-arterial therapeutic concept constitutes only one of many technologies to facilitate drug delivery to the brain and brain tumors. An enhanced understanding of the BBB physiology has led to the development of a multitude of noninvasive and invasive strategies to target tumor cells present beyond this barrier. Locoregional invasive technologies, in particular, are a remarkably fast-evolving segment within the neurooncology field. These technologies are based on local delivery of therapeutics directly to the brain, thereby bypassing the BBB entirely and facilitating smaller initial drug dosage and minimal systemic absorption. They include drug delivery to the cerebrospinal fluid via intrathecal or intraventricular injections and interstitial delivery via biodegradable polymers or catheters [17]. Diffusion-based approaches such as intracavitary wafers placed at the time of tumor resection, intrathecal injection using the Ommaya reservoir, and intraventricular injection via lumbar puncture are limited by the restricted tissue penetrance of most therapeutic agents into structures not immediately adjacent to the brain surface, hindering them from reaching deep and infiltrative tumor cells [21,27,117]. However, molecularly engineered cells, especially chimeric antigen receptor (CAR) T-cells and natural killer cells, feature enhanced tumor-homing abilities and have shown promise in preclinical and clinical investigations of these strategies in various brain tumors [118,119,120,121,122,123,124,125,126]. In contrast to locoregional therapies, intra-arterial administration takes advantage of the branched blood vessel system that is feeding into brain tumors, thereby reaching even distant, infiltrative tumor cells, and enabling site-directed infiltration of cell-based therapies or diffusion of macromolecules [127].

Direct interstitial drug infusion to brain tumors is achieved by placing one or multiple catheters under stereotactic guidance into the bulk tumor. These cannulas can be connected to osmotic or mechanical pumps and allow for direct, targeted delivery [28,29]. The former can provide continuous drug delivery at a set infusion rate, whereby the infusion is driven by an osmotic pressure gradient [128]. Convection-enhanced delivery (CED), which describes direct interstitial infusion under a mechanical pressure gradient, has further advantages, including a larger, more homogeneous volume of drug distribution [129]. This technology is currently being used in multiple clinical trials for brain tumors like glioblastoma and diffuse intrinsic pontine glioma (DIPG) [130,131,132,133,134,135,136,137,138]. In addition to technical and procedural challenges, such as catheter design and placement, tracking of infusate distribution, prevention of reflux along the canula tract, and reduction in edema and mechanical tissue damage, the success of CED is hampered by the potential requirement of repetitive infusions [139,140]. Almost 100 clinical trials for DIPG have failed, and it has recently been shown that the brain half-life of panobinostat, a small molecule inhibitor, after CED is only 2.9 h [141,142]. Intra-arterial technologies address some of these pitfalls, allowing for repeated and prolonged therapeutic administration to ensure pharmacologic effect, but are accompanied by their own constraints, as discussed above. Consequently, a “one size fits all” approach may not be effective, and a rational combination of therapeutics and their delivery strategies should be considered. In this way, a comprehensive treatment regime to attain optimal concentrations in brain tumors over a prolonged period of time while minimizing off-target effects could be established.

There are certain limitations that are inherent to bibliometric and clinical trials analyses. First, Elsevier’s Scopus and ClinicalTrials.gov are only two of many databases for articles and clinical trials, respectively. With both of them being US-based, this may have influenced the predominance of US-based literature and trials in this study. There is validity to the argument that our representation of the research landscape of intra-arterial brain tumor therapy would have been more comprehensive if additional registries would have been included. Even though both Scopus and ClinicalTrials.gov constitute major databases in their respective field, holding registrations from journals and institutions around the world, there is a distinct possibility that this study is an underestimate of all articles and trials evaluating intra-arterial brain tumor treatments. Second, since our therapy type and treatment strategy classifications were solely based on the literature and our own experience, it is unclear whether the presented categories and proportions optimally reflect the current status and trends of the field. It is possible that more refined categorizations would have led to a better representation. Finally, article and clinical trial parameters, e.g., citation count and number of institutions involved, are regularly updated to rigorously reflect the current state of affairs. As it is difficult to continue updating these parameters once they have been extracted, our study represents the status quo and trends of articles and clinical trials on intra-arterial drug delivery for brain tumor treatment as of August 2021. We anticipate that analyses in the future will confirm whether these were accurate.

## 5. Conclusions

In this bibliometric and clinical trials analysis, we identified, characterized, and analyzed available parameters of preclinical and clinical research on intra-arterial therapy for brain tumors. Overall, 271 articles and 20 clinical trials were sufficiently specific for inclusion. Among articles, most were CL and chemotherapy was the most common therapeutic modality. With respect to treatment strategies for optimizing intra-arterial drug delivery, transient blood–brain barrier disruption using mannitol was the most frequently studied. These trends were reflected in clinical trials, but unfortunately only a single phase 1/phase 2 study has reported outcomes to date. Given the longstanding history of intra-arterial brain tumor therapy research, our results mandate the consideration of novel therapeutic and procedural strategies, including precision medicine, nanoparticles, and superselective intra-arterial cerebral infusion, to foster the preclinical research basis and set the stage for more robust, systematic clinical trials in the future.

## Figures and Tables

**Table 1:** Summary of article characteristics.

Parameter	Outcome (*n* = 271 Publications) *
Publication Type	
Original articles	227 (84%)
Review articles	44 (16%)
Clinical articles	201 (74%)
Basic science articles	70 (26%)
Open access	54 (20%)
Year of publication	
Range in years	1962–2021
Peak year	1986
Number of publications in peak year	17
Median publications per year	5
Citations	
Median	15
Most cited publication (*n*)	Primary central nervous system lymphoma (607)
Most cited original article (*n*)	Safety and efficacy of a multicenter study using intraarterial chemotherapy in conjunction with osmotic opening of the blood–brain barrier for the treatment of patients with malignant brain tumors (300)
Most cited review article (*n*)	Primary central nervous system lymphoma (607)
Authors	
Median number of authors per publication	5
Most authored publications (*n*)	Neuwelt E.A. (14)
Most first authored publications (*n*)	Nakagawa H. (7)
Most senior authored publications (*n*)	Neuwelt E.A. (8), Boockvar J.A. (8)
Country of correspondence	
Total countries involved	20
Countries with most publications	
US	135 (50%)
Japan	46 (17%)
Canada	19 (7%)
Contributing journals	
Total number of journals involved	120
Journals with most publications	
*Journal of Neuro-Oncology*	48 (18%)
*Japanese Journal of Cancer and Chemotherapy*	14 (5%)
*Neurosurgery*	13 (5%)
Tumor type #	
Most common	
Glioma (combined)	184 (68%)
Multiple (>1 tumor type)	66 (24%)
Therapies #	
Chemotherapy	215 (79%)
Targeted Therapy	40 (15%)
Immunotherapy	13 (5%)
Radiosensitizing/neutron capture therapy	17 (6%)
Stem cell therapy	5 (2%)
Treatment strategies #	
Number of publications using:	
Nanoparticles	17 (6%)
Transient blood–brain barrier disruption	74 (27%)
Transient cerebral hypoperfusion or flow arrest	6 (2%)
Superselective intra-arterial cerebral infusion	27 (10%)
Imaging techniques with contrast or labelled therapeutic agents	13 (5%)

* Categorical data reported as *n* (% total). # Does not sum to 271 as studies could report more than one tumor type and therapeutic approach.

**Table 2:** Summary of clinical trial characteristics.

Parameter	Outcome (*n* = 20 Trials) *
Time (expected)	
Start year	2011 (1998–2019)
Completion year	2022 (2008–2025)
Results first posted	2015 ^
Last updated	2020 (2013–2021)
Status	
Current status as of August 2021	
Recruiting	8 (40%)
Completed	6 (30%)
Suspended	2 (10%)
Active, not recruiting	2 (10%)
Terminated	1 (5%)
Unknown status	1 (5%)
Study results available	1 (5%)
Cohort	
Minimum age of enrollment (years)	18 (0–18)
Maximum age of enrollment (years)	99 (17–120)
Design	
Interventional studies	20 (100%)
Phase	
Phase 1	9 (45%)
Phase 1 + Phase 2	8 (40%)
Phase 2	3 (15%)
Outcomes #	
Primary	
Safety and toxicity	11 (55%)
PFS	6 (35%)
OS	5 (25%)
Secondary	
PFS	11 (55%)
Safety and toxicity	9 (45%)
OS	8 (40%)
QOL	5 (25%)
Location and funding	
Two most common corresponding institutes	
Northwell Health	10 (50%)
OHSU Knight Cancer Institute	3 (15%)
Trials per Country	
US	18 (90%)
China, Canada	1 (5%) each
Number of sites involved	1 (1–2)
Therapies #	
Number of research studies using:	
Targeted Therapy	13 (65%)
Chemotherapy	8 (40%)
Treatment mechanism #	
Number of research studies using:	
Superselective intra-arterial cerebral Infusion ^1^	13 (65%)
Transient blood–brain barrier disruption using mannitol ^2^	12 (60%)
Three most common conditions #	
Glioblastoma	13 (65%)
Anaplastic Astrocytoma	8 (40%)
Brain Metastasis	3 (15%)

PFS, progression-free survival; OS, overall survival; QOL, quality of life. * Continuous data reported as the median (range) and categorical data reported as *n* (% total). ^ Only 1 trial. # Does not sum to 20 as trials could report more than one condition, outcome, and therapeutic approach. ^1^ Superselective intra-arterial cerebral infusion into a major tumor feeding artery was performed using neurovascular microcatheter systems under fluoroscopic guidance to increase the concentration of drug delivered to the tumor while sparing the patient of systemic side effects. ^2^ Temporary opening of the blood–brain barrier was achieved by treating patients with an intra-arterial infusion of the osmotic agent mannitol followed by intra-arterial administration of therapeutic agents (mannitol 20–25%; 3–12.5 mL over 2 min).

**Table 3:** Summary of clinical trial with reported results.

Parameter	Outcome
NCT number	NCT00362817
Title	Carboplatin and Temozolomide (Temodar) for Recurrent and Symptomatic Residual Brain Metastases
Location	Ohio State University, Columbus, Ohio, United States
Start date	October 2004
Finish date	January 2008
Results first posted	May 2015
Enrolment size	17
Age range (years)	18 and older
Primary outcome	
Response rate	42.8%
Secondary outcome	
Overall survival in weeks	25.2
Time to progression in weeks (mean)	22.6
Incidence of CNS toxicities	0
Cause of death	CNS tumor = 0, systemic disease progression = 7

## Data Availability

All data supporting reported results can be found in the manuscript and supplementary materials to the manuscript.

## Supplementary Materials

The following are available online at https://www.mdpi.com/article/10.3390/pharmaceutics13111885/s1, Table S1. Articles on intra-arterial therapy for brain tumors based on publication type; Table S2. Classification of tumors investigated in articles on intra-arterial therapy for brain tumors; Table S3. Citations for articles on intra-arterial therapy for brain tumors; Table S4. Authors of articles on intra-arterial therapy for brain tumors; Table S5. Years of publications of intra-arterial therapy for brain tumors differentiated by article type; Table S6. Involvement of countries in articles on intra-arterial therapy for brain tumors; Table S7. Involvement of journals in articles on intra-arterial therapy for brain tumors; Table S8. Investigated chemotherapies and application ways in articles on intra-arterial therapy for brain tumors; Table S9. Investigated targeted therapies in articles on intra-arterial therapy for brain tumors; Table S10. Investigated immunotherapies in articles on intra-arterial therapy for brain tumors; Table S11. Investigated radiosensitizing/neutron capture therapies in articles on intra-arterial therapy for brain tumors; Table S12. Investigated stem cell therapies in articles on intra-arterial therapy for brain tumors; Table S13. Investigated treatment strategies in articles on intra-arterial therapy for brain tumors; Table S14. Tumor types investigated in clinical trials on intra-arterial therapy for brain tumors; Table S15. Timeline of clinical trials on intra-arterial therapy for brain tumors, including start year, completion year, year of the first release of results, and last updated year; Table S16. Recruitment status of clinical trials on intra-arterial therapy for brain tumors; Table S17. Number of patients enrolled in clinical trials intra-arterial therapy for brain tumors; Table S18. Minimum and maximum age of enrollment in clinical trials on intra-arterial therapy for brain tumors; Table S19. Distribution of the different study phases of clinical trials on intra-arterial therapy for brain tumors; Table S20. Type of primary intervention investigated by the clinical trials on intra-arterial therapy for brain tumors; Table S21. Detailed primary interventions applied by the clinical trials on intra-arterial therapy for brain tumors; Table S22. Therapies investigated in clinical trials on intra-arterial therapy for brain tumors; Table S23. Treatment strategies investigated in clinical trials on intra-arterial therapy for brain tumors; Table S24. Different outcome measurements of clinical trials on intra-arterial therapy for brain tumors with respect to primary and secondary outcome, overall survival, progression-free survival, safety aspects, and quality of life; Table S25. Sponsors, institutions and countries involved in clinical trials on intra-arterial therapy for brain tumors.
