# Supplementary material for: Status Quo and Trends of Intra-Arterial Therapy for Brain Tumors: A Bibliometric and Clinical Trials Analysis
Source: Pharmaceutics. 2021 Nov 6;13(11):1885. doi: 10.3390/pharmaceutics13111885 (PMC8625566; doi:10.3390/pharmaceutics13111885)
Supplement: Supplementary file 1 [file pharmaceutics-13-01885-s001.zip › pharmaceutics-1408064-supplementary.pdf]

pharmaceutics

MDPI

Supplementary Materials: Status Quo and Trends of Intra-Arterial Therapy for Brain Tumors: A Bibliometric and Clinical Trials Analysis

Julian S. Rechberger, Frederic Thiele and David J. Daniels

Table S1. articles on intra-arterial therapy for brain tumors based on publication type.

Publication Type	Freq.	Percent
Original articles	227	84%
Clinical articles	201	74%
Reviews	44	16%
Basic articles	70	26%
Open Access Studies	54	20%

Table S2. classification of tumors investigated in articles on intra-arterial therapy for brain tumors.

Tumor type		
Total tumor types	10	
**Tumor types in all publications**	**Freq.**	**Percent**
Gliomas	155	57,2%
Multiple (> 1 tumor type)	66	24,35%
Glioblastoma	17	6,27%
Brain metastasis	12	4,43%
Gliosarcoma	10	3,69%
Lymphoma	5	1,85%
Diffuse intrinsic pontine glioma	2	0,74%
Ependymoma	2	0,74%
CNS leukemia	1	0,37%
Osteogenic sarcoma	1	0,37%
**Tumor types in Articles**	**Freq.**	**Percent**
Gliomas	137	60,35%
Multiple (> 1 tumor type)	47	20,7%
Glioblastoma	15	6,61%
Metastases	11	4,85%
Gliosarcoma	10	4,41%
Lymphoma	3	1,32%
CNS leukemia	1	0,44%
Osteogenic sarcoma	1	0,44%
Ependymoma	1	0,44%
Diffuse intrinsic pontine glioma	1	0,44%
**Tumor types in Reviews**	**Freq.**	**Percent**
Gliomas	18	40,91%
Multiple (> 1 tumor type)	19	43,18%
Glioblastoma	2	4,55%
Lymphoma	2	4,55%
Diffuse intrinsic pontine glioma	1	2,27%

Metastases	1	2,27%
Ependymoma	1	2,27%

Table S3. citations for articles on intra-arterial therapy for brain tumors.

**Citations**	
Median citations per publication	15 (0-607)
Most cited paper (n)	Primary central nervous system lymphoma (607)
Most cited article (n)	Safety and efficacy of a multicenter study using intraarterial chemotherapy in conjunction with osmotic opening of the blood-brain barrier for the treatment of patients with malignant brain tumors (300)
Most cited review (n)	Primary central nervous system lymphoma (607)

Table S4. authors of articles on intra-arterial therapy for brain tumors.

**Authors**	
Median number of authors per publication	5 (1-18)
Most authored publications	Neuwelt E.A. (14), Bookvar J.A. (10), Greenberg H.S. (10)
Most authored articles	Neuwelt E.A. (14), Greenberg H.S. (10), Nakagawa H. (8)
Most authored reviews	Bookvar J.A. (3), Burkhardt J.-K. (3)
Most first authored publications (n)	Nakagawa H. (7)
Most first authored articles (n)	Nakagawa H. (6), Greenberg H.S. (6)
Most first authored reviews (n)	Newton H.B. (2), Hochberg F.H. (2), Joshi S. (2)
Most senior authored publications (n)	Neuwelt E.A. (8), Boockvar J.A. (8)
Most senior authored articles (n)	Neuwelt E.A. (8)
Most senior authored reviews (n)	Boockvar J.A. (3)

Table S5. years of publications of intra-arterial therapy for brain tumors differentiated by article type.

**Year of publication**	
Range in years	1962 - 2021
Peak year	1986
Number of publications in peak year	17
Median publications per year	5
Peak year for basic science publications	1999 (6)
Peak year for clinical article publications	1986 (16)
Peak year for original articles	1986 (17)
Peak year for review articles	2020 (4)

Table S6. involvement of countries in articles on intra-arterial therapy for brain tumors.

**Country of correspondence**	**Freq.**	
Number of countries involved	20	

Publications per country	Freq.	Percent
US	135	49,82%
Japan	46	16,97%
Canada	19	7,01%
France	12	4,43%
China	11	4,06%
Germany	8	2,95%
Spain	5	1,85%
Norway	4	1,48%
Italy	4	1,48%
Australia	2	0,74%
Austria	2	0,74%
Taiwan	2	0,74%
Brazil	1	0,37%
United Kingdom	1	0,37%
Slovakia	1	0,37%
South Korea	1	0,37%
India	1	0,37%
Netherlands	1	0,37%
Israel	1	0,37%
Finland	1	0,37%
NA	13	4,8%
**Articles per country**	**Freq.**	**Percent**
US	110	48,46%
Japan	45	19,82%
Canada	16	7,05%
China	7	3,08%
France	7	3,08%
Germany	6	2,64%
Spain	5	2,2%
Norway	4	1,76%
Austria	2	0,88%
Australia	2	0,88%
Italy	2	0,88%
Taiwan	2	0,88%
Israel	1	0,44%
Brazil	1	0,44%
South Korea	1	0,44%
Slovakia	1	0,44%
United Kingdom	1	0,44%
Finland	1	0,44%
NA	13	5,73%
**Reviews per country**	**Freq.**	**Percent**
US	25	56,82%
France	5	11,36%
China	4	9,09%
Canada	3	6,82%
Germany	2	4,55%
Italy	2	4,55%

Japan	1	2,27%
India	1	2,27%
Netherlands	1	2,27%

Table S7. involvement of journals in articles on intra-arterial therapy for brain tumors.

**Contributing journals**	**Freq.**	
Total number of journals involved	120	
**Most common journals**	**Journal**	**Freq.**
Original articles	Journal of Neuro-Oncology	45
Reviews	Journal of Neuro-Oncology	3
Basic science articles	Journal of Neuro-Oncology	13
Clinical articles	Journal of Neuro-Oncology	35
**Contributing journals overall**	**Freq.**	**Percent**
Journal of Neuro-Oncology	48	17,71%
Japanese Journal of Cancer and Chemotherapy	14	5,17%
Neurosurgery	13	4,8%
Journal of Neurosurgery	11	4,06%
Cancer	9	3,32%
Cancer Research	9	3,32%
American Journal of Neuroradiology	8	2,95%
Neurologia Medico-Chirurgica	6	2,21%
Cancer Treatment Reports	5	1,85%
Neurological Surgery	4	1,48%
Interventional Neuroradiology	4	1,48%
Cancer Chemotherapy and Pharmacology	3	1,11%
Journal of NeuroInterventional Surgery	3	1,11%
Neuro-Oncology	3	1,11%
Revue Neurologique	3	1,11%
Acta Neurochirurgica	3	1,11%
Surgical Neurology	3	1,11%
The Journal of neuroscience nursing: journal of the American Association of Neuroscience Nurses	3	1,11%
Cancer Gene Therapy	3	1,11%
Gene Therapy	3	1,11%
Neuroradiology	2	0,74%
Cancer Drug Delivery	2	0,74%
International Journal of Radiation Oncology Biology Physics	2	0,74%
Journal of Experimental Therapeutics and Oncology	2	0,74%
European Journal of Cancer	2	0,74%

American Journal of Clinical Oncology: Cancer Clinical Trials	2	0,74%
World Neurosurgery	2	0,74%
Neurology	2	0,74%
Journal of Nuclear Medicine	2	0,74%
Clinical Cancer Research	2	0,74%
Anticancer Research	2	0,74%
Gan to kagaku ryoho. Cancer & chemotherapy	2	0,74%
Acta radiologica. Supplementum	2	0,74%
Neuroimaging Clinics of North America	1	0,37%
Lymphokine and Cytokine Research	1	0,37%
Current Clinical Pharmacology	1	0,37%
Current oncology reports	1	0,37%
Journal of Neuroradiology	1	0,37%
Journal of Clinical Neuroscience	1	0,37%
Current Treatment Options in Oncology	1	0,37%
Current Cancer Drug Targets	1	0,37%
Journal of Neurosurgical Anesthesiology	1	0,37%
Oncotarget	1	0,37%
Neurological Research	1	0,37%
Oncology Letters	1	0,37%
Photochemistry and Photobiology	1	0,37%
Frontiers in Bioscience	1	0,37%
CNS Drugs	1	0,37%
Frontiers in Oncology	1	0,37%
International Journal of Molecular Sciences	1	0,37%
Anti-Cancer Drugs	1	0,37%
Update on Cancer Therapeutics	1	0,37%
Cancers	1	0,37%
Neurochirurgie	1	0,37%
Journal for ImmunoTherapy of Cancer	1	0,37%
Journal of Virology	1	0,37%
Journal of Experimental and Clinical Cancer Research	1	0,37%
Quarterly Journal of Nuclear Medicine	1	0,37%
Clinical Genitourinary Cancer	1	0,37%
Acta Neurochirurgica, Supplement	1	0,37%
American Journal of Roentgenology	1	0,37%
Journal of Drug Targeting	1	0,37%
Pharmaceutical Research	1	0,37%
CANCER CHEMOTHER.REP.	1	0,37%
The Israel Medical Association journal : IMAJ	1	0,37%
Chinese journal of integrated traditional and Western medicine	1	0,37%
International Journal of Cancer	1	0,37%
International Journal of Radiation Oncology, Biology, Physics	1	0,37%
Drug Delivery and Translational Research	1	0,37%
Clinical and Translational Oncology	1	0,37%
Human Gene Therapy	1	0,37%

Shikoku Acta Medica	1	0,37%
Neurologia medico-chirurgica	1	0,37%
ONCOLOGY (United States)	1	0,37%
Radiology	1	0,37%
International Journal of Hyperthermia	1	0,37%
Selective Cancer Therapeutics	1	0,37%
British Medical Journal	1	0,37%
Cancer Investigation	1	0,37%
Archives of Neurology	1	0,37%
Journal of Clinical Oncology	1	0,37%
Brain and Nerve	1	0,37%
Nuclear Medicine Communications	1	0,37%
Pharmaceutics	1	0,37%
Neurosurgery Clinics of North America	1	0,37%
Canadian Journal of Neurological Sciences	1	0,37%
Bailliere's Clinical Neurology	1	0,37%
European Neurology	1	0,37%
Current Problems in Cancer	1	0,37%
Cancer Journal from Scientific American	1	0,37%
Journal of Neurosurgery: Pediatrics	1	0,37%
Current Opinion in Oncology	1	0,37%
Neurologic Clinics	1	0,37%
Cancer Bulletin	1	0,37%
Pathologie Biologie	1	0,37%
Journal of neurosurgery	1	0,37%
Investigational New Drugs	1	0,37%
Bone Marrow Transplantation	1	0,37%
Drug Delivery: Journal of Delivery and Targeting of Therapeutic Agents	1	0,37%
Neurologia	1	0,37%
Expert Review of Anticancer Therapy	1	0,37%
Bulletin du Cancer	1	0,37%
Chinese journal of surgery	1	0,37%
Journal of the National Cancer Institute	1	0,37%
Oncology Reports	1	0,37%
Canadian Association of Radiologists Journal	1	0,37%
Investigative Radiology	1	0,37%
Canadian Journal of Neurological Sciences / Journal Canadien des Sciences Neurologiques	1	0,37%
Vestnik khirurgii imeni I. I. Grekova	1	0,37%
Journal of Clinical Neuro-Ophthalmology	1	0,37%
Revista da Associacao Medica Brasileira	1	0,37%
Chinese Medical Journal	1	0,37%
Acta Oncologica	1	0,37%
Journal of Practical Oncology	1	0,37%
Advances and technical standards in neurosurgery	1	0,37%
Recent results in cancer research	1	0,37%
Annals of the New York Academy of Sciences	1	0,37%
Radiotherapy and Oncology	1	0,37%

Radiation and Environmental Biophysics	1	0,37%
Journal of Pediatric Hematology/Oncology	1	0,37%

Table S8. investigated chemotherapies and application ways in articles on intra-arterial therapy for brain tumors.

Chemotherapy	Freq.	Percent
Total	215	100%
i.a. carmustine	29	13,49%
general	23	10,7%
i.a. nimustine	20	9,3%
i.a. cisplatin	15	6,98%
multiple	14	6,51%
i.a. carboplatin	12	5,58%
unspecified	11	5,12%
i.a. methotrexate	7	3,26%
i.a. cisplatin + carmustine	6	2,79%
i.a. cisplatin + etoposide	6	2,79%
i.a. HeCNU	5	2,33%
i.a. temozolomide	4	1,86%
i.a. etoposide	4	1,86%
i.a. carmustine + systemic vincristine + procarbazine	3	1,4%
i.a. cisplatin + nimustine	3	1,4%
i.a. carmustine + nimustine	3	1,4%
i.a. adriamycin	3	1,4%
i.a. cisplatin + systemic carmustine	3	1,4%
i.a. carboplatin + systemic etoposide	2	0,93%
i.a. cisplatin + carboplatin	2	0,93%
systemic carboplatin	2	0,93%
i.a. epodyl	2	0,93%
i.a. MCNU	2	0,93%
i.a. Docetaxel	1	0,47%
i.a. adriamycin + 5FU	1	0,47%
i.a. nimustine + systemic UFT + PSK	1	0,47%
i.a. carboplatin + etoposide + systemic cytoxan	1	0,47%
i.a. nimustine + streptozotocin	1	0,47%
i.a. O6BG + systemic carmustine	1	0,47%
i.a. AZQ	1	0,47%
i.a. alkylators	1	0,47%
i.a. SarCNU	1	0,47%
i.a. PCNU	1	0,47%
i.a. carmustine + systemic DFMO	1	0,47%
4-HC	1	0,47%
i.a. vincristine	1	0,47%
systemic temozolomide	1	0,47%
systemic methotrexate	1	0,47%
systemic carboplatin + cyclophosphamide + etoposide	1	0,47%
i.a. carmustine + systemic 5-FU	1	0,47%
i.a. carmustine + systemic methotrexate	1	0,47%

i.a. cisplatin + carmustine + teniposide + systemic cisplatin + teniposide + cytosine arabinoside	1	0,47%
i.a. nimustine + systemic phenobarbital	1	0,47%
i.a. neocarzinostatin	1	0,47%
i.a. carmustine + nimustine + HeCNU	1	0,47%
i.a. carmustine + FUdR + methotrexate	1	0,47%
i.a. carmustine + PCB	1	0,47%
i.a. carmustine + PV	1	0,47%
i.a. mitoxantrone	1	0,47%
i.a. melphalan	1	0,47%
i.a. cisplatin + systemic etoposide	1	0,47%
i.a. cisplatin + systemic PCNU	1	0,47%
i.a. cisplatin + adriamycin + mitomycin C	1	0,47%
i.a. cisplatin + bleomycin	1	0,47%
i.a. cisplatin + carboplatin + oxaliplatin	1	0,47%
i.a. endoxan + trenimon + arsenic	1	0,47%
i.a. cisplatin + systemic spiromustine	1	0,47%

Table S9. investigated targeted therapies in articles on intra-arterial therapy for brain tumors.

Targeted Therapy	Freq.	Percent
Total	40	100%
General	11	27,5%
Bevacizumab	7	17,5%
Bevacizumab + cetuximab	3	7,5%
HSV hrr3	2	5%
Cetuximab	2	5%
Adenovirus containing p53	1	2,5%
Adenovirus mutant thymidine kinase	1	2,5%
Cintredekin	1	2,5%
Genetic vectors	1	2,5%
Elemene	1	2,5%
Endostatin	1	2,5%
HSV + lacz	1	2,5%
HSV thymidine kinase	1	2,5%
HSV replication conditional	1	2,5%
Hsv g47δ	1	2,5%
Kringle 1-5	1	2,5%
Lacz 2157	1	2,5%
Peptide trans-activator of transcription (TAT)	1	2,5%
Trans-activator of transcription	1	2,5%
Adenovirus	1	2,5%

Table S10. investigated immunotherapies in articles on intra-arterial therapy for brain tumors.

Immunotherapy	Freq.	Percent
Total	13	100%
General	5	38,46%
TNF-alpha	5	38,46%
TALL-104 cells	1	7,69%
IFN-gamma	1	7,69%

IFN-beta	1	7,69%

Table S11. investigated radiosensitizing/neutron capture therapies in articles on intra-arterial therapy for brain tumors.

Radiosensitizing/neutron capture therapy	Freq.	Percent
Total	17	100%
BUdR	5	29,41%
BPA	3	17,65%
BSH + BPA	3	17,65%
BSH	2	11,76%
General	1	5,88%
IUdR	1	5,88%
Multiple	1	5,88%
Photofrin	1	5,88%

Table S12. investigated stem cell therapies in articles on intra-arterial therapy for brain tumors.

Stem cell therapy	Freq.	Percent
Total	5	100%
MSCs	3	60%
Hematopoietic stem cells	1	20%
Endothelial cells	1	20%

Table S13. investigated treatment strategies in articles on intra-arterial therapy for brain tumors.

Treatment strategies	Freq.	Percent
Number of publications including at least 1 treatment strategy	104	38,37%
**Nanoparticles**	**Freq.**	**Percent**
Total	17	100%
Liposomal	8	47,06%
General	5	29,41%
Mono crystalline iron oxide nanoparticles	2	11,76%
Magnetic microspheres	1	5,88%
Neutral micelles	1	5,88%
**Transient blood-brain barrier disruption**	**Freq.**	**Percent**
total	74	100%
mannitol	48	64,86%
bradykinin/ RMP-7	16	21,62%
unspecified	7	9,46%
focused ultrasound	2	2,7%
papaverine	1	1,35%
**Transient cerebral hypoperfusion or flow arrest**	**Freq.**	**Percent**
Total	6	2,21%
**Superselective intra-arterial cerebral infusion (SIACI)**	**Freq.**	**Percent**
Total	27	9,96%
**Imaging techniques with contrast or labeled therapeutic agents**	**Freq.**	**Percent**
Total	13	4,8%

Table S14. tumor types investigated in clinical trials on intra-arterial therapy for brain tumors.

**Condition**	**Freq.**	Percent
Glioblastoma	13	65%
Anaplastic Astrocytoma	8	40%
Brain Metastasis	3	15%
Malignant Glioma	3	15%
Brain and Central Nervous System Tumor	2	10%
Diffuse Intrinsic Pontine Glioma	2	10%
Malignant Germ Cell Tumor	1	5%
Primitive Neuroectodermal Tumor	1	5%
Pilomyxoid Astrocytoma	1	5%
Mixed Oligodendroglioma-Astrocytoma	1	5%
Medulloepithelioma	1	5%
Medulloblastoma	1	5%
Gliosarcoma	1	5%
Lymphoma	1	5%
Brain Stem Glioma	1	5%
Anaplastic Oligoastrocytoma	1	5%
Germ Cell Tumor	1	5%
Fibrillary Astrocytoma	1	5%
Embryonal Tumor With Multilayered Rosettes	1	5%
Central Nervous System Embryonal Neoplasm	1	5%
Vestibular Schwannoma	1	5%

Table S15. timeline of clinical trials on intra-arterial therapy for brain tumors, including start year, completion year, year of the first release of results, and last updated year.

**Timeline**		
Start year	Freq.	Percent
2019	1	5%
2018	1	5%
2016	2	10%
2015	1	5%
2014	1	5%
2013	3	15%
2011	2	10%
2010	2	10%
2009	3	15%
2008	1	5%
2004	1	5%
2003	1	5%
1998	1	5%
Completion	Freq.	Percent
2025	1	5%
2023	1	5%
2022	10	50%
2021	1	5%
2020	1	5%
2018	1	5%
2016	2	10%

2014	1	5%
2012	1	5%
2008	1	5%
Results first posted year	Freq.	Percent
NA	19	95%
2015	1	5%
Last updated year	Freq.	Percent
2021	6	30%
2020	7	35%
2019	1	5%
2018	1	5%
2017	3	15%
2015	1	5%
2013	1	5%

Table S16. recruitment status of clinical trials on intra-arterial therapy for brain tumors.

**Status**	**Freq.**	Percent
Recruiting	8	40%
Completed	6	30%
Suspended	2	10%
Active, not recruiting	2	10%
Terminated	1	5%
Unknown status	1	5%

Table S17. number of patients enrolled in clinical trials intra-arterial therapy for brain tumors.

**Enrollment number**	**Freq.**	Percent
30	4	20%
17	2	10%
21	2	10%
54	2	10%
25	1	5%
33	1	5%
37	1	5%
15	1	5%
48	1	5%
60	1	5%
3	1	5%
36	1	5%
47	1	5%
35	1	5%

Table S18. minimum and maximum age of enrollment in clinical trials on intra-arterial therapy for brain tumors.

**Age of enrollment**		
Minimum age	Freq.	Percent
18 years	18	90%
1 years	1	5%
1 month	1	5%
Maximum age (years)	Freq.	Percent

99	15	75%
75	1	5%
120	1	5%
21	1	5%
17	1	5%
45	1	5%

Table S19. distribution of the different study phases of clinical trials on intra-arterial therapy for brain tumors.

**Phases**	**Freq.**	Percent
Phase 1	9	45%
Phase 1 + Phase 2	8	40%
Phase 2	3	15%

Table S20. type of primary intervention investigated by the clinical trials on intra-arterial therapy for brain tumors.

**Primary intervention**	**Freq.**	Percent
Drug alone	14	70%
Drug+Radiation	2	10%
Drug+Other	2	10%
Biological alone	1	5%
Biological+Drug	1	5%

Table S21. detailed primary interventions applied by the clinical trials on intra-arterial therapy for brain tumors.

**Detailed primary intervention**	**Freq.**	Percent
i.a. Bevacizumab (Avastin)	5	25%
i.a. Cetuximab	3	15%
i.a. Melphalan	2	10%
i.a. Trastuzumab	1	5%
i.a. Carboplatin + oral Temozolomide	1	5%
i.a. Carboplatin + systemic Cyclophosphamide + systemic Etoposide phosphate	1	5%
i.a. Bevacizumab (Avastin) + i.a. Cetuximab (Erbitux)	1	5%
i.a. Temozolomide	1	5%
i.a. Bevacizumab (Avastin) + i.a. Carboplatin	1	5%
i.a. Oncolytic Adenovirus Ad5-DNX-2401	1	5%
i.a. ADV-TK/GCV	1	5%
i.a. Carboplatin	1	5%
i.a. Carboplatin + i.a. Melphalan + systemic sodium thiosulfate	1	5%

Table S22. therapies investigated in clinical trials on intra-arterial therapy for brain tumors.

**Therapy**		
Chemotherapy	Freq.	Percent
Y	8	40%
N	12	60%
Targeted therapy	Freq.	Percent

Y	13	65%
N	7	35%
Immunotherapy	Freq.	Percent
Y	0	0%
N	20	100%
Stem cell or bone marrow transplant	Freq.	Percent
Y	0	0%
N	20	100%
Hormone therapy	Freq.	Percent
Y	0	0%
N	20	100%

Table S23. treatment strategies investigated in clinical trials on intra-arterial therapy for brain tumors.

**Nanoparticles**	**Freq.**	Percent
Y	0	0%
N	20	100%
Transient blood-brain barrier disruption	Freq.	Percent
Y	12	60%
N	8	40%
IA-TCH or IA-FA	Freq.	Percent
Y	0	0%
N	20	100%
SIACI	Freq.	Percent
Y	13	65%
N	7	35%
Imaging techniques with contrast or labeled therapeutic agents	Freq.	Percent
Y	0	0%
N	20	100%

Table S24. different outcome measurements of clinical trials on intra-arterial therapy for brain tumors with respect to primary and secondary outcome, overall survival, progression free survival, safety aspects, and quality of life.

**Outcome measurements**		
Overall survival	Freq.	Percent
Secondary	8	40%
NA	7	35%
Primary	5	25%
Progression free survival	Freq.	Percent
Secondary	11	55%
Primary	6	30%
NA	3	15%
Feasibility/safety/toxicity	Freq.	Percent
Primary	10	50%
Secondary	8	40%
NA	1	5%
Primary + Secondary	1	5%
Quality/activities of daily living/functional status	Freq.	Percent

NA	15	75%
Secondary	5	25%
Other	Freq.	Percent
NA	13	65%
Secondary (Response Evaluation Criteria in Solid Tumors)	1	5%
Secondary (Tumor response + blood count)	1	5%
Secondary (Efficacy of chemotherapy)	1	5%
Secondary (Tumor response + virus replication + virus shedding)	1	5%
Secondary (clinical benefit)	1	5%
Secondary (Tumor response)	1	5%
Primary (Tumor response + intracellular carboplatin accumulation)	1	5%

Table S25. sponsors, institutions and countries involved in clinical trials on intra-arterial therapy for brain tumors.

**Locations**		
Sponsors	Freq.	Percent
Northwell Health	10	50%
OHSU Knight Cancer Institute	3	15%
Ohio State University Comprehensive Cancer Center	1	5%
Weill Medical College of Cornell University	1	5%
Sidney Kimmel Comprehensive Cancer Center at Johns Hopkins|Solving Kids' Cancer	1	5%
M.D. Anderson Cancer Center|DNAtrix, Inc.	1	5%
Huazhong University of Science and Technology|Beijing Tiantan Hospital|Beijing Chao Yang Hospital|Beijing Friendship Hospital	1	5%
Global Neurosciences Institute	1	5%
Centre hospitalise universitaire de Sherbrooke	1	5%
Corresponding institution	Freq.	Percent
Lenox Hill Brain Tumor Center	10	50%
OHSU Knight Cancer Institute	3	15%
Ohio State University	1	5%
Weill Cornell Medical College/New York Presbyterian Hospital	1	5%
The Johns Hopkins Hospital	1	5%
M D Anderson Cancer Center	1	5%
Beijing YouAn Hospital	1	5%
Philadelphia	1	5%
CHUS	1	5%
Country	Freq.	Percent
US	18	90%
China	1	5%
Canada	1	5%
